# Transcriptomic profiles reveal differences in zinc metabolism, inflammation, and tight junction proteins in duodenum from cholesterol gallstone subjects

**DOI:** 10.1038/s41598-020-64137-7

**Published:** 2020-05-04

**Authors:** Eleodoro Riveras, Lorena Azocar, Tomas C. Moyano, Marcia Ocares, Hector Molina, Diego Romero, Juan C. Roa, Jose R. Valbuena, Rodrigo A. Gutiérrez, Juan F. Miquel

**Affiliations:** 1 Departamento de Gastroenterología. Facultad de Medicina. Pontificia Universidad Católica de Chile, Santiago, Chile; 2 Departamento de Anatomía Patológica. Facultad de Medicina. Pontificia Universidad Católica de Chile, Santiago, Chile; 3 Millennium Institute for Integrative Biology, iBio. FONDAP Center for Genome Regulation. Departamento de Genética Molecular y Microbiología. Facultad de Ciencias Biológicas. Pontificia Universidad Católica de Chile, Santiago, Chile

**Keywords:** Cholelithiasis, Transcriptomics, Genetics research

## Abstract

Cholesterol Gallstone Disease (GSD) is a common multifactorial disorder characterized by crystallization and aggregation of biliary cholesterol in the gallbladder. The global prevalence of GSD is ~10–20% in the adult population but rises to 28% in Chile (17% among men and 30% among women). The small intestine may play a role in GSD pathogenesis, but the molecular mechanisms have not been clarified. Our aim was to identify the role of the small intestine in GSD pathogenesis. Duodenal biopsy samples were obtained from patients with GSD and healthy volunteers. GSD status was defined by abdominal ultrasonography. We performed a transcriptome study in a discovery cohort using Illumina HiSeq. 2500, and qPCR, immunohistochemistry and immunofluorescence were used to validate differentially expressed genes among additional case-control cohorts. 548 differentially expressed genes between GSD and control subjects were identified. Enriched biological processes related to cellular response to zinc, and immune and antimicrobial responses were observed in GSD patients. We validated lower transcript levels of metallothionein, NPC1L1 and tight junction genes and higher transcript levels of genes involved in immune and antimicrobial pathways in GSD patients. Interestingly, serum zinc and phytosterol to cholesterol precursor ratios were lower in GSD patients. A significant association was observed between serum zinc and phytosterol levels. Our results support a model where proximal small intestine plays a key role in GSD pathogenesis. Zinc supplementation, modulation of proximal microbiota and/or intestinal barrier may be novel targets for strategies to prevent GSD.

## Introduction

Cholesterol Gallstone Disease (GSD) is a common multifactorial disorder characterized by cholesterol crystal formation, precipitation and growth (stones) in the gallbladder^1^. World prevalence is 10–20% for the adult population^2^. However, the Chilean population has the highest prevalence of GSD in the world, it being 17% among men and 30% among women^1,3^. Many risk factors for GSD development have been identified such as ethnic background, advanced age, female gender, family history and genetic susceptibility^4,5^. The diagnosis of GSD is established by visualization of macroscopic gallstones through an abdominal ultrasound. Surgery (cholecystectomy), which is the only effective form of treatment available, represents a significant health burden in countries with high prevalence^6^. GSD is associated with common metabolic conditions such as obesity, insulin resistance, diabetes mellitus, hypertriglyceridemia and pregnancy^7^. In the context of western diet and lifestyle^8,9^ and the higher prevalence of GSD and the consequent health burden, it is critical to understand pathogenic mechanisms of this complex disease.

Intestinal mucosa allows the absorption of nutrients, electrolytes and water, while also serving as an effective defense that limits systemic contamination by intraluminal microbes or their products^10^. In particular, lipid absorption (sterols and triglycerides) take place in the proximal small intestine (duodenum and jejunum). Evidence suggests that intestinal cholesterol absorption is a risk factor of GSD disease and inhibiting it with ezetimibe can prevent GSD formation, at least in animal models^11,12^. Changes in gut microbiota has been associated with GSD in animal models and humans^13–15^. Diet can significantly affect the species composition of gut microbiota and several immune disorders like IBD, allergies and asthma^16–18^. Interestingly, the duodenum shares the same embryological origin as the liver and biliary tree and seems to have coordinated regulation of gene expression^19^. Therefore, the overall impact of the intestine in gallstone formation may at least partly explain the molecular mechanisms involved in GSD pathogenesis.

To explore the potential role of the upper small intestine in the pathogenesis of GSD, we performed transcriptome analysis of duodenal mucosa from selected cholesterol gallstone subjects and matched controls. We validated our findings using independent case-control cohorts.

## Results

### The human duodenal transcriptome in GSD pathologies

**Figure 1:**
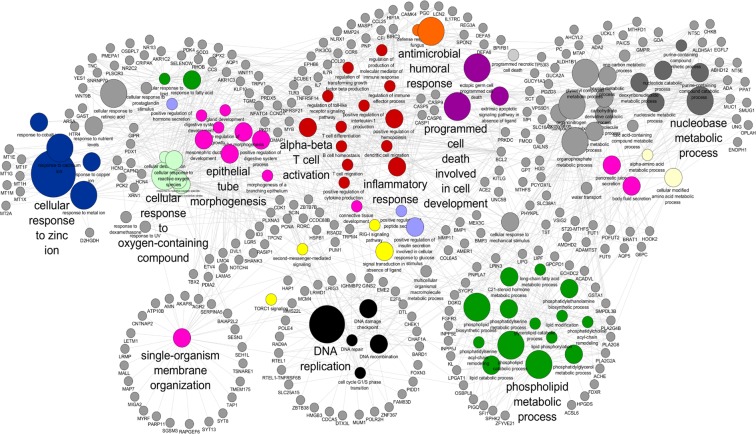
Network of gene ontology (GO) terms enriched in the human duodenal transcriptome of cholesterol gallstone patients. The enriched biological processes predicted from differential gene expression between control and GSD patients were grouped with the software ClueGO v2.3 (http://www.ici.upmc.fr/cluego/) as a functional cluster (using a kappa score = 0.3). Biological processes and enrichment significance terms are represented as nodes and node size, respectively. Associated genes are represented as dots. Edges represent term-gene interaction. Community cluster via GLay plugin in Cytoscape was applied in determining modules with functional properties.

Finely coordinated hepatobiliary and gastrointestinal function is crucial to prevent GSD formation^12^. However, the contribution of the small intestine in GSD pathogenesis has not been well-studied. As a first step to determine the role of the small intestine in GSD, we analyzed global gene expression in duodenal mucosa in five selected GSD patients and four paired control subjects using RNA sequencing (discovery cohort, Table 1). We identified 548 differentially expressed genes (DEGs) between GSD and control subjects using DESeq. 2 analysis with a false discovery rate (FDR) cutoff of ≤ 0.05 (Table S1). 168 genes were induced and 380 genes were repressed in GSD. ClueGO was used to facilitate identification of biological processes associated with these differentially expressed genes^20^ (Fig. 1). The ClueGO network allows for visualizing and clustering gene ontology and pathway terms that participate in the same biological function to identify relationships between differentially expressed genes and enriched biological process. The main enriched biological processes were cellular response to zinc, epithelial tube morphogenesis, antimicrobial and immune responses, apoptosis, DNA replication, and phospholipid metabolic processes (Fig. 1).

**Table 1:** Clinical characteristics of discovery and validation cohorts from GSD and control subjects.

Variable	Discovery			First validation cohort			Second validation cohort		
	Control (n = 4)	GSD (n = 5)	P value	Control (n = 24)	GSD (n = 24)	P value	Control (n = 34)	GSD (n = 25)	P value
Age	25.78 ± 2.09	24.57 ± 5.16	ns	32.16 ± 8.15	31.16 ± 7.60	ns	25.5 ± 7.30	23.68 ± 3.79	ns
BMI (Kg/m^2^)	27.25 ± 4.99	27.80 ± 2.17	ns	24.36 ± 3.84	25.39 ± 3.94	ns	23.97 ± 3.05	24.31 ± 3.53	ns
Female gender (%)	100	100	ns	100	100	ns	100	100	ns
Total Cholesterol (mg/dL)	185.25 ± 24.87	167.6 ± 25.26	ns	172.54 ± 28.25	179.29 ± 34.83	ns	157.18 ± 31.88	163.33 ± 23.79	ns
HDL Cholesterol (mg/dL)	63.25 ± 10.43	44.8 ± 10.77	0.036	57.79 ± 12.87	52 ± 13.57	ns	54.61 ± 12.84	47.44 ± 12.03	0.033
LDL Cholesterol (mg/dL)	101.5 ± 26.75	101.6 ± 23.28	ns	95.67 ± 25.29	106.96 ± 26.73	ns	85.38 ± 24.99	95.24 ± 22.52	ns
Triglycerides (mg/dL)	102.5 ± 53.16	103.2 ± 42.03	ns	95.54 ± 54.38	99.67 ± 52.16	ns	85.91 ± 45.66	85.41 ± 32.39	ns
Glucose (mg/dL)	74.5 ± 1.73	78 ± 7.38	ns	77 ± 6.09	81.29 ± 6.68	ns	79.91 ± 8.69	81.56 ± 6.86	ns
Insulin (mg/dL)	5.38 ± 1.75	10.34 ± 4.23	ns	8.32 ± 5.03	8.55 ± 3.38	ns	11.16 ± 11.97	11.37 ± 5.41	ns
HOMA_IR_	0.99 ± 0.34	1.96 ± 0.70	0.041	1.59 ± 0.98	1.72 ± 0.68	ns	2.23 ± 2.42	2.28 ± 1.10	ns

ns = no significant difference between GSD and control subjects.

Differential gene expression and functional analysis showed biological processes involved in cellular response to zinc ions, DNA replication and phospholipid metabolic processes were repressed, while antimicrobial and immune responses and apoptosis were overexpressed in GSD subjects (Table S1 and S2).

### Serum zinc status in association with intestinal cholesterol absorption may contribute to pathophysiological mechanism of GSD

**Figure 2:**
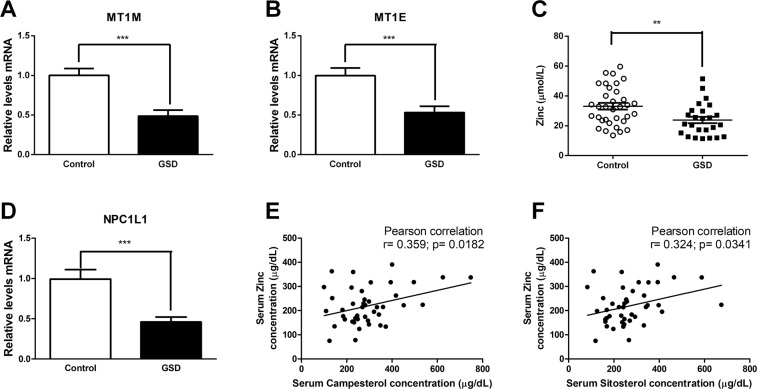
Cellular response to zinc, serum zinc levels and sterol absorption are altered in cholesterol gallstone diseases. Duodenal biopsies and serum were obtained from healthy volunteers (white bars) and GSD patients (black bars). A, B, D) MT1M, MT1E and NPC1L1 gene expression of healthy volunteers (n = 24) and patients with GSD (n = 24) was evaluated by qPCR. C) Serum zinc concentrations were quantified using total reflection X-ray fluorescence (TXRF), and E-F) serum phytosterol of healthy volunteers (N = 34) and GSD patients (N = 25) was quantified with GC-MS. Gene 18 S was used as a normalizer. *p < 0.05 and ***p < 0.001. MT1M, Metallothionein 1M and MT1E, Metallothionein 1E.

In order to confirm the role of cellular response to zinc in GSD, we first validated changes in gene expression identified by RNA sequencing using real time PCR in RNA samples obtained from duodenal mucosa from the first validation cohort of 24 control and 24 GSD patients (Table 1). We assayed two genes associated with cellular response to zinc ions (metallothionein 1 M, MT1M and metallothionein 1E, MT1E). Both genes were significantly down-regulated in GSD subjects, which was consistent with the RNA sequencing data (Fig. 2A,B). Metallothionein expression is induced by a zinc-dependent transcription factor that binds to the promoter region of the metallothionein gene^21^. This suggests that mRNA abundance of metallothionein in the duodenal mucosa of GSD subjects is an indication of altered zinc status. To evaluate the zinc status in GSD pathologies, we assessed the serum zinc concentration in a second validation cohort (Table 1) using total reflection X-ray fluorescence (TXRF) spectrometry. As Fig. 2C shows, GSD subjects had significantly lower levels of serum zinc than control subjects, which is consistent with the lower mRNA abundance of metallothionein in duodenal mucosa of GSD subjects.

A moderate level of zinc deficiency has been reported in many gastrointestinal disorders such as malabsorption syndromes, liver diseases, Crohn´s disease, and regional ileitis^22,23^. However, the relationship between zinc levels and GSD is unknown. It is known that GSD subjects display increased biliary cholesterol output and lower intestinal cholesterol absorption^24^. We observed that the duodenal mucosa of GSD patients is lower than that of controls in mRNA levels of NPC1L1 and serum levels of surrogate markers of cholesterol absorption (Fig. 2D, Figure S1). Interestingly, a positive correlation was found between zinc and phytosterol serum levels (Fig. 2E,F), which suggests that physiological regulation of zinc levels is relevant to GSD pathogenesis and the relationship between zinc levels and serum surrogate markers of cholesterol absorption is relevant to understanding the associated phenotype of low cholesterol absorption and higher synthesis in GSD subjects^24^.

### Patients with GSD have elevated antimicrobial and immune duodenal responses

**Figure 3:**
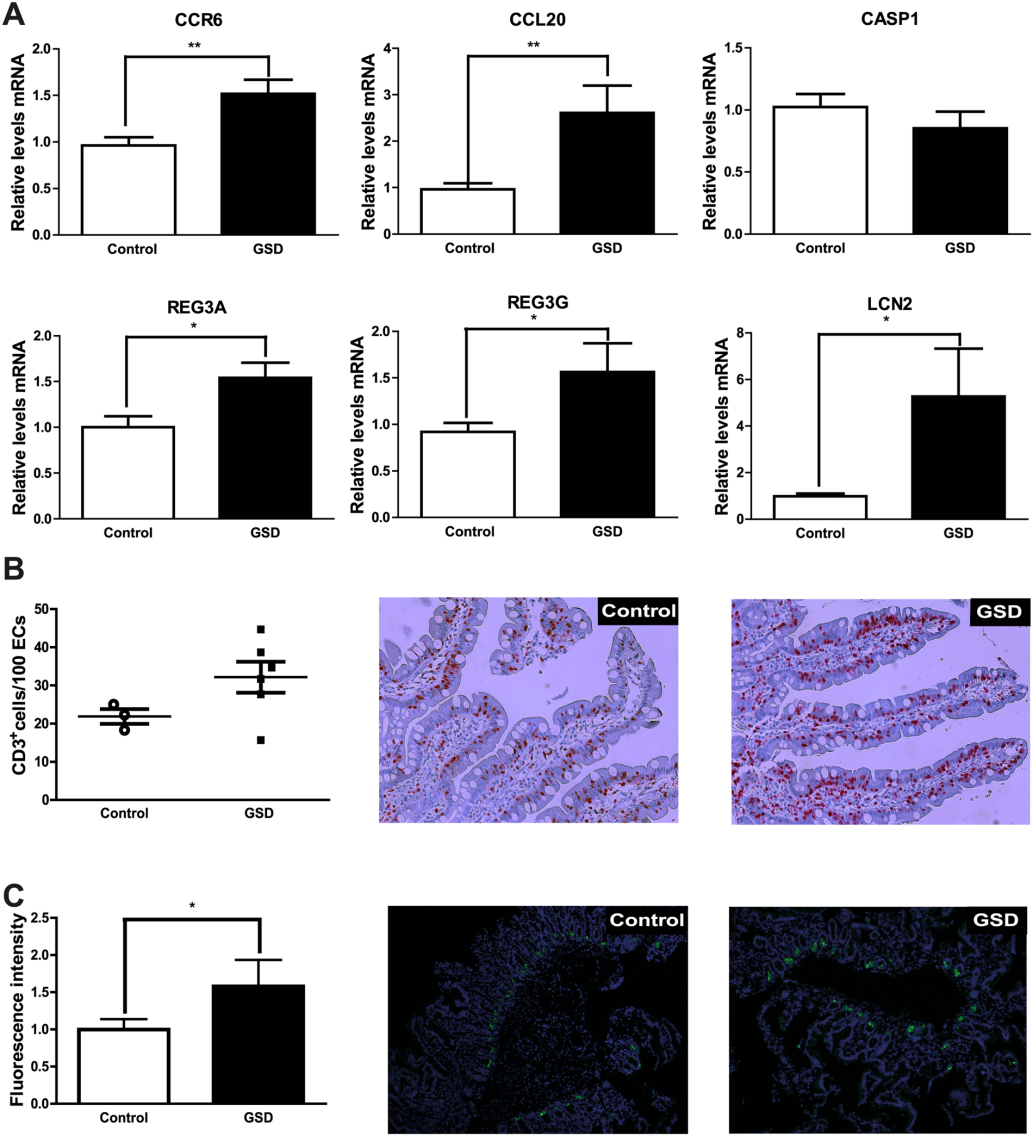
Intestinal immune and antimicrobial responses are enhanced in cholesterol gallstone diseases. Duodenal tissue was obtained by biopsy from healthy volunteers (white bars) and GSD patients (black bars). A) The expression of the genes CCR6, CCL20, CASP1, REG3A, REG3G, and LCN2 of healthy volunteers (n = 24) and GSD patients (n = 24) was evaluated by qPCR. B) CD3-immunohistochemical staining and C) Lysozyme-immunofluorescence staining evaluations of formalin-fixed paraffin-embedded tissues samples from duodenal mucosa of GSD (n = 6) and control subjects (N = 3). *p < 0.05 and **p < 0.01.

Three antimicrobial and three immune genes were selected to confirm the role of antimicrobial and immune responses in duodenal mucosa of GSD subjects (Tables S2 and 1). Of the 6 genes, we only observed over-expression of antimicrobial (REG3G, LCN2, DEFA6) and immune (CCR6 and CCL20) genes in GSD as compared to control subjects (Fig. 3A). We selected these genes for validation because they are associated with inflammatory response, restricting bacterial colonization and leading monocyte chemotaxis to intestinal epithelium^25^. To test whether the over-expressed genes are associated with low-grade inflammation, we further assessed intraepithelial lymphocytes (IEL) counts and lysozyme expression by immunohistochemical and immunofluorescent analysis in formalin-fixed paraffin-embedded tissue samples from duodenal mucosa of GSD and control subjects. The IEL counts and lysozyme expression were significantly higher in the duodenal mucosa of GSD subjects than in that of control subjects (Fig. 3B,C). However, the increase of lysozyme expression was independent of Paneth cell abundance in GSD (Figure S3). This result suggests duodenal mucosa of GSD subjects exhibits alterations to the intestinal immune system and antimicrobial response consistent with the dysbiosis observed in the gut microbiota of GSD subjects^14,26^.

Despite a significant correlation (r = 0.92, p < 0.0001, Figure S2) between RNA sequencing and real-time PCR values, we could not validate genes associated with epithelial tube morphogenesis, apoptosis, DNA replication, and phospholipid metabolic process in GSD patients (Figure S4). This may be due to the dynamic nature of transcriptome expression or variation in gene expression among individuals.

### Expression of duodenal tight junction proteins is deregulated in GSD subjects

**Figure 4:**
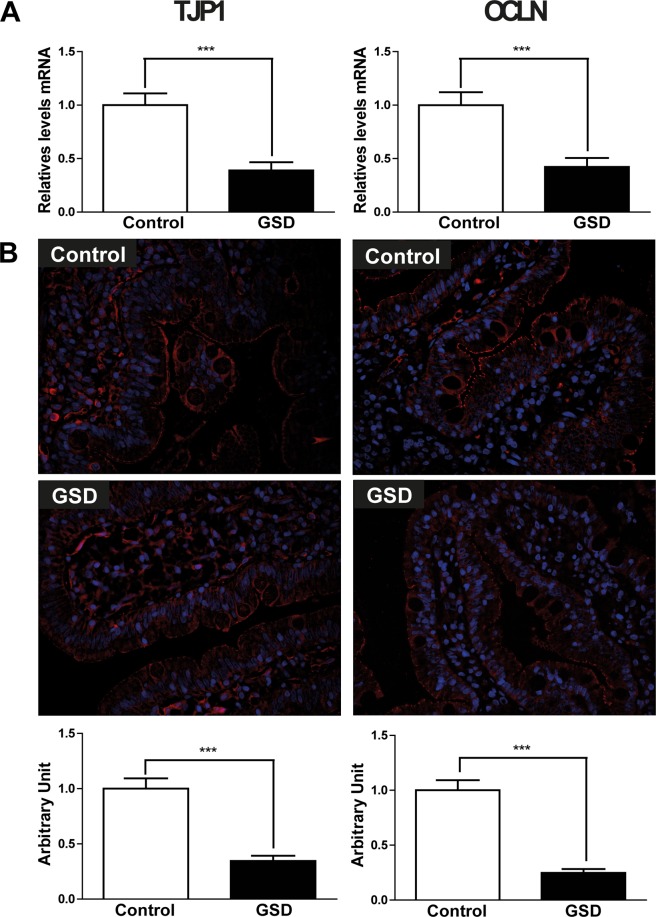
Tight junction gene expressions are decreased on duodenal biopsy of cholesterol gallstone patients. Gene expression, immunofluorescence and densitometry analysis of TJP1 (left) and OCLN (right) of biopsied duodenal tissue: A) TJP1 and OCLN gene expression of healthy volunteers (n = 24, white bars) and GSD patients (N = 24, black bars) was evaluated by qPCR. B) TJP1 and OCLN protein expression were quantified in formalin-fixed paraffin-embedded tissue samples from duodenal mucosa of GSD and control subjects by measuring the average fluorescent intensity in 10 non-overlapping fields per subject by microscopy. Data are the mean of three control subjects and six patients with GSD. ***p < 0.001.

A plausible explanation for the lower serum zinc level, low-grade inflammation, and enhanced antimicrobial response in the duodenal mucosa of GSD patients is perturbation of the intestinal barrier integrity due to alteration of the tight junction proteins^10,27,28^. To evaluate the integrity of the tight junction in the proximal gut of GSD patients, we analyzed gene expression of the tight junction protein 1 (TJP1) and occluding junction (OCLN) of the first validation cohort using Real-Time PCR. Both genes were downregulated in GSD subjects in contrast to those of control subjects (Fig. 4A). We further evaluated protein expression levels of both genes by immunofluorescence assays in formalin-fixed paraffin-embedded tissues samples from duodenal mucosa of GSD and control subjects to validate our findings. The GSD subjects were confirmed to have significantly lower expression levels of tight junction proteins than the controls (Fig. 4A,B). These results indicate that intestinal barrier integrity is deregulated in the context of the GSD phenotype.

## Discussion

While biliary cholesterol hypersecretion is the first step in GSD pathogenesis, downstream events (i.e. biliary tree, gut) are required for cholesterol stone formation in the gallbladder^1^. The small Intestine has been implicated in the susceptibility to diseases within the gut (i.e. IBD, coeliac disease) or distal target organs (i.e. allergies, NASH, stress)^10^. However, its contribution to GSD pathogenesis have not been well studied^1,12^. We found 548 DEGs between GSD and control subjects. Among GSD patients, we observed enriched biological processes related to cellular response to zinc, immune and antimicrobial responses, epithelial tube morphogenesis, apoptosis, DNA replication, and phospholipid metabolic process, and others. In a second step, we validated some of these results in an independent cohort of cases and controls. Consistent with the RNA sequencing analysis, metallothionein genes were down-regulated in GSD subjects in association with lower serum zinc levels than those of control patients. In addition, patients with GSD are characterized by enhanced expression of genes related to antimicrobial and immune processes, intestinal intraepithelial lymphocytes counts and expression of lysozyme proteins, indicating the presence of low-grade inflammation and enhanced antimicrobial responses in the duodenal mucosa of GSD patients. Furthermore, GSD subjects have low levels of tight junction proteins, which indicates that intestinal barrier integrity is altered. The alteration of duodenal mucosal integrity and antimicrobial and immune responses suggests bacterial-mucosal interactions and might be associated with gut microbiota dysbiosis, which is reported in GSD patients^13–15^.

Zinc is an essential trace element for growth, development, DNA and RNA synthesis, and immunity, and is involved in many gastrointestinal disorders^29^. Zinc homeostasis is primarily regulated by intestinal absorption, renal reabsorption, and clearance of endogenous zinc in the intestine (fecal excretion) through both pancreatic and liver excretion^30^. Moderate serum zinc deficiency and low metallothionein expression in duodenal mucosa observed in GSD patients may be due to lower dietary availability, low gastrointestinal absorption or increased zinc loss. However, the mechanism involved in zinc status in GSD subjects has not been studied. Interestingly, we observed that SLC39A11/ZIP11 was the only zinc transporter significantly regulated in GSD using RNA sequencing (Table S4). ZIP11 is a zinc transporter expressed in testis and the digestive system^31^ and may play a role in zinc import when zinc is deficient. ZIP11 protein levels has been found to be reduced in the stomach and elevated in the colon during zinc depletion in mice^32^. This suggests that dietary zinc deficiency regulates ZIP11 expression, and points to the need for research into the role of dietary zinc supplementation in GSD formation. Several studies have shown that zinc supplementation has an anti-inflammatory effect and reduces alteration of intestinal permeability^30^, indicating that zinc supplementation is a potential strategy to prevent cholesterol gallstone formation.

The development of GSD is mainly caused by hypersecretion of hepatic cholesterol into bile^1^. However, cholesterol homeostasis in human is finely regulated by intestinal absorption, endogenous biosynthesis, and biliary and fecal excretion of cholesterol^12,33^. Intestinal sterol absorption and sterol secretion are respectively mediated primarily by NPC1L1 and ABCG5/8 dimers. Interestingly, the low level of NPC1L1 gene expression in duodenal mucosa correlates with the low serum phytosterol levels in GSD patients. Our results show a positive correlation between serum zinc levels and serum phytosterol levels, which suggests that low serum zinc levels are associated with low intestinal sterol absorption. Interestingly, low intestinal sterol absorption has a negative correlation with an increase of cholesterol excretion and biosynthesis in GSD^24^. Genetic studies have shown that polymorphism in ABCG5/8 transporters are associated with GSD and a gain of function in biliary cholesterol secretion^34^. Also, in Chinese gallstone patients have been observed an increase of ABCG5 and ABCG8 gene expression in the liver^35^. Therefore, a lower intestinal sterol absorption mediated by NPC1L1 and an increase in sterol excretion mediated by ABCG5/ABCG8 are consistent with a reduced phytosterol levels in serum.

Interestingly, it has been reported that zinc depletion in rats and humans significantly reduces total serum cholesterol, principally because of lower levels of high-density lipoprotein-cholesterol (HDL-C)^36,37^. Consistently, many studies have shown that low HDL cholesterol concentrations significantly correlates with GSD^3,38,39^. Interestingly, the overexpression of scavenger receptor class B type 1 (SR-BI), which mediates the hepatic uptake and clearance of cholesterol from HDL, has been associated with GSD pathogenesis in murine models of GSD^40^. In addition, Chinese gallstone patients have an increase expression of SRBI protein in the liver which might contribute to the hypersecretion of HDL-cholesterol derived into the bile^35^. Clearly, the relationship between zinc levels, low HDL cholesterol and GSD may be complex and need further study.

The gut microbiome has emerged as a key factor in many gastrointestinal disorders like Crohn’s disease, ulcerative colitis and coeliac disease^41–43^. Recent studies have shown that gut bacterial translocation allows some microbiota to reach the biliary tree in some scenarios. Indeed, biliary microbiota can promote gallstones formation in model mice and possibly in humans^13,14,26,44^. The observed dysfunction of intestinal barrier integrity, in conjunction with higher IEL counts (intraepithelial lymphocytes), elevated lysozyme protein and antimicrobial peptide expression in GSD patients strongly suggests that dysbiosis of gut microbiome contributes to GSD pathogenesis. However, the underlying mechanism between GSD pathogenesis and gut microbiome remains unclear and deserves further investigation.

The limitation of the present study, as with any clinical study of associations, is that we cannot state definitively whether our findings precede and constitute risk factors for gallstone formation or are secondary events. It is possible that chronic exposure of the proximal gut to lithogenic bile (cholesterol supersaturated bile and biliary bacterial factors) leads to at least some of the observed changes in gene and protein expression profiles^15,26,45,46^. It is also possible that changes in the biliary composition modify proximal gut microbiota, leading to changes in the intestinal barrier and local low-grade inflammation response. A further longitudinal study using healthy individuals carrying D19H polymorphism in ABCG5/8 might clarify whether our findings are cause or consequence of gallstone formation.

In conclusion, the transcriptome approach allowed us to characterize the global gene expression of the upper small intestine of GSD subjects for the first time. Using two independent cohorts of cases and control, we showed that GDS subjects have lower serum zinc levels, enhanced immune and antimicrobial responses, and disrupted intestinal barrier function. These molecular changes in duodenal mucosa and zinc metabolism may be contributing factors to cholesterol gallstone pathogenesis. However, further exploration and functional characterization of this biological process are needed to identify new strategies to prevent and treat cholesterol gallstone disease.

### Statement of significance

What is already known about this subject?

- Intestinal cholesterol absorption is a risk factor in GSD.
- Gut microbiota has been associated with GSD.

What are the new findings?

- Patients with GSD display enriched biological processes in cellular response to zinc, and antimicrobial and immune responses.
- Gallstone disease patients have lower expression levels of key genes involved in zinc metabolism in the proximal gut in association with lower serum zinc levels.
- Antimicrobial and immune response pathways are enhanced in the proximal gut of gallstone disease patients.
- Lower expression levels of the tight junction proteins suggest the existence of enhanced mucosal permeability in the proximal gut of gallstone disease patients.

How might it impact on clinical practice in the foreseeable future?

- Our transcriptomic and physiological approaches indicate that serum zinc status, microbiota and intestinal mucosal integrity contribute to pathophysiological mechanisms of GSD. Zinc supplementation, and modulation of microbiota and the intestinal barrier may be novel strategies to prevent cholesterol gallstone formation.

## Material and Method

### Study design and participants

We carried out a whole transcriptome assay using a discovery cohort and two independent cohorts of case and control subjects to validate our findings. The discovery group consisted of 5 cholesterol gallstone patients and 4 matched controls. These individuals were recruited from the Gastroenterology and Endoscopic Unit of the Red Salud UC-Christus, Pontificia Universidad Católica de Chile. Patients that were carriers of gallstones, as determined by abdominal ultrasound, and that met the inclusion criteria (standardized interview and clinical evaluation) were invited to participate in this study by agreeing to provide blood samples for biochemical tests and to undergo upper endoscopy with duodenal biliary drainage. The inclusion criteria were: women between 18 and 35 years old, non-obese (BMI 18–29 kg/m^2^), with no disease other than being an asymptomatic GSD carrier two months prior to the study. Additional inclusion criteria were at least two months without intake of medications such as non-steroidal anti-inflammatory drugs, aspirin, antibiotics, metformin or lipid lowering drugs, not pregnant and normal blood test results for glycemia, biochemical profile and negative serological test for celiac disease. Examination of duodenal bile after the stimulation of gallbladder contraction with 20% amino acid solution (20 ml endoluminal) showed cholesterol crystals under light microscopy, indicative of cholesterol gallstones^47^. Subjects with gastroesophageal reflux or dyspepsia that were referred for an upper endoscopy and with a normal abdominal ultrasound were invited to participate in the study as controls. Duodenal drainage was not performed with the control group. Biopsy samples were taken from the second segment of the duodenum (distal to the ampulla of Vater) in aseptic conditions and were stored in RNA-latter solution for RNA sequencing and real-time reverse transcriptase PCR (RT-PCR).

A first validation cohort consisted of 24 GSD and 24 control subjects, prospectively recruited from the same Gastroenterology Department of the Red Salud UC-Christus, Pontificia Universidad Católica de Chile, and meeting the same inclusion criteria as the discovery group. The presence or absence of GSD was defined by abdominal ultrasound. Biopsy samples were taken from the second segment of the duodenum during an upper endoscopy and were used for real-time reverse transcriptase PCR (RT-PCR) or fixed in formalin and embedded in paraffin for immunofluorescence and immunohistochemical assays.

A second validation cohort was a nested case-control study selected from a recent population-based study described elsewhere to identify genetic and metabolic risk factors for GSD^48^. Biobank serum from this cohort were available at the Gastroenterology Department of the Pontificia Universidad Católica de Chile. The selected group consisted of 34 controls and 25 GSD patients that met the same inclusion criteria as the discovery group. The presence or absence of GSD was defined by abdominal ultrasound. Serum samples were used to assess serum zinc and non-cholesterol sterol concentrations.

All subjects signed informed consent forms and the protocol was approved by the Institutional Review Board for Human Studies at Pontificia Universidad Católica de Chile and were conducted in accordance with the guidelines of the National Commission on Science and Technology (CONICYT-Chile).

### RNA-seq analysis

To understand the role of the upper small intestine in the molecular mechanism involved in GSD pathogenesis, we employed a transcriptomic approach with RNA sequencing to evaluate changes in the gene expression profile in the upper gut of GSD subjects. Whole transcriptome data were generated from duodenal mucosal biopsy samples of 5 GSD patients and 4 matched control individuals. A PureLink RNA Mini Kit (Ambion Life technologies, Carlsbad, USA) was used for total RNA preparation. Sequencing was conducted with the TruSeq Stranded mRNA Library Preparation Kit (Illumina, San Diego, CA). The prepared libraries were sequenced on an Illumina HiSeq. 2500. The samples were sequenced to a depth of 9 samples per lane, which generated ~18 million 50 bp single-end reads per sample. We used HISAT2^49^ for alignment with the human genome and Rsubread^50^ for the read count per transcript. The DESeq. 2 package v1.26 (https://bioconductor.org/packages/DESeq. (2)^51^ was used to capture differentially expressed genes, with an adjusted false discovery rate P -value of 0.05.

### Gene enrichment analysis

A functional analysis was conducted with ClueGO v2.3 (http://www.ici.upmc.fr/cluego/)^20^, a plug-in of Cytoscape^52^, to identify enriched biological processes from the list of differentially expressed genes (DEG) between control and GSD subjects. A functionally grouped network of enriched biological process terms was generated from the DGE list. The statistical criterion was a two-sided (enrichment/depletion) hyper-geometric distribution test with a p-value significance level of ≤ 0.05. The Kappa-statistic score threshold was set at 0.3 and GO levels was set at 4 to 6. Cluster analysis with clusterMaker2 plugin^53^ provided a comprehensive view of node connectivity. Community clustering (GLay) determined the modules with functional properties.

### Gene expression analysis

Total RNA from duodenal mucosal biopsies was extracted with the PureLink RNA Mini Kit (Ambion Life technologies, Carlsbad, USA) and reverse-transcribed into cDNA with a High-Capacity cDNA Reverse Transcription Kit (Thermo Fisher Scientific Inc., Carlsbad, USA). Real-time RT-PCR was performed using System Brillant III Ultra-Fast SyBR Green QPCR Master Mix (Agilent technology) and human primers (Supplementary Table 3) on a StepOne Real-Time PCR System (Thermo Fisher Scientific). The results were normalized to the human 18 S ribosomal RNA gene. The data were normalized to 1 with respect to the healthy control group. The statistical significance was determined with a two-tailed t-test (*P < 0.05).

### Serum zinc concentration

Serum samples were obtained from the second validation cohort and stored at −80 °C in a serum biobank at the Pontificia Universidad Católica de Chile. The inclusion criteria were the same as those noted above. Hemolyzed samples were excluded. Serum zinc concentration was determined by total reflection x-ray fluorescence (TXRF) spectrometry using an S2 PICOFOX spectrometer (Bruker, Germany). The samples were prepared in simple 1:5 dilutions of serum:ultrapure water. Galio (Ga) was added for internal standardization. After homogenization, the solution was transferred to a quartz glass sample carrier and dried in a desiccator for 10 minutes. All samples were measured at an X-ray excitation of 50 kV/750 µA for 600 seconds.

### Measurement of non-cholesterol sterols

The serum concentration of phytosterols (campesterol and sitosterol) and cholesterol precursors (lathosterol and desmosterol) were measured by gas chromatography/mass spectrometry (GC/MS) as described elsewhere^24,54^. The GC/MS were done with an Agilent 6890 N gas chromatograph with a 5973 network mass selective detector (Agilent technologies) equipped with an HP-5ms capillary column (30 m × 0.25 mm × 0.25 µm) (Agilent Technologies).

### Immunofluorescence

Two-micron sections of duodenal biopsies were deparaffinized and blocked following general procedures and incubated overnight at 4 °C with rabbit anti-ZO-1 (61–7300) and Occludin Antibody (PA5–20755) (1:100, Thermo Fisher Scientific). The secondary antibody used was goat anti-rabbit Alexa Fluor 594 (1:500, ab150080, Abcam). Microscopy images of 5 representative non-overlapping fields were obtained with Nikon Eclipse Ni microscope equipped with the software NIS-Element Freeware v2.3 (https://www.microscope.healthcare.nikon.com). TJP1 and OCLN expression were quantified by measuring the average fluorescence intensity in 10 non-overlapping fields per subject using Image Pro (WaveMetrics, Oregon, Washington, USA).

### Immunohistochemistry

Paraffin embedded tissue sections in positively charged slides were deparaffinized and blocked following the same protocols mentioned above. Slides were incubated overnight at 4 °C with antibodies against CD3 (A045201–2, DAKO). Immunostaining was performed with an ultraView Universal DAB Detection Kit (760–500, Ventana Medical Systems, Basal, Switzerland) using BenchMark ULTRA instruments (Ventana Medical Systems), according to the manufacturer´s recommendations. Intraepithelial lymphocytes (IELs) were determined by counting at least 300 epithelial cells (ECs). Only lymphocytes above the basal membrane were regarded as IELs. Counts were expressed as the number of IELs per 100 ECs.

### Statistical analysis

The population was stratified into two groups: Control and GSD. The results of the quantitative determinations were expressed as the mean ± standard deviation (SD). Differences between the groups were analyzed using a Student’s t-test with a 95% confidence level of significance. A Pearson’s correlation test was applied to determine relationships among the variables. Values of p < 0.05 were considered statistically significant.

## Supplementary information

Supplementary information.

Supplementary Table S1.

Supplementary Table S2.
