# Supplementary material for: Transcriptomic profiles reveal differences in zinc metabolism, inflammation, and tight junction proteins in duodenum from cholesterol gallstone subjects
Source: Sci Rep. 2020 May 4;10:7448. doi: 10.1038/s41598-020-64137-7 (PMC7198580; doi:10.1038/s41598-020-64137-7)
Supplement: Supplementary file 1 — Supplementary information. [file 41598_2020_64137_MOESM1_ESM.docx]

**Transcriptomic profiles reveal differences in zinc metabolism, inflammation, and tight junction proteins in duodenum from cholesterol gallstone subjects**

E. Riveras^1^, L. Azocar^1^, TC. Moyano^3^, M. Ocares^1^, H. Molina^1^, D. Romero^2^, JC. Roa^2^, JR. Valbuena^2^, RA. Gutiérrez^3^, JF. Miquel^1^.

**Figure S1. Ratio of serum phytosterols to cholesterol precursors and ABCG5 and ABCG8 gene expression in control and GSD patients.**

A) Serum phytosterols and cholesterol precursors from healthy volunteers (N=34) and GSD patients (N=25) were quantified using GC-MS. Results are expressed as the ratios phytosterol to cholesterol precursors as surrogate index of cholesterol absorption and synthesis. B-C) Pearson correlation between serum zinc concentration and phytosterols/cholesterol precursors ratio. D-E) ABCG5 and ABCG8 gene expression was evaluated by qPCR in healthy volunteers (n=24) and patients with GSD (n=24). White dots represent healthy volunteers and black dots represent GSD patients. *18S* gene was used as normalizer. *p<0.05 and **p<0.01.





**Figure S2. Correlation between RNA sequencing and real time PCR data on differentially gene expressed.**

Pearson correlation were performed using log2 fold change values from RNA-Seq and qPCR analysis. 24 genes were selected for comparison between RNA-Seq and qRT-PCR.


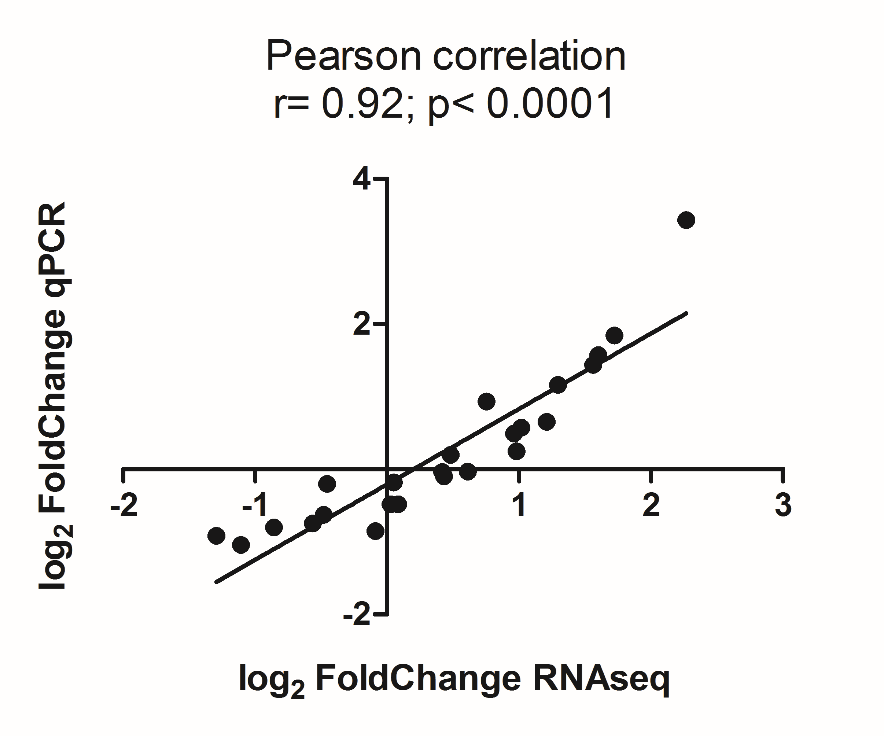


**Figure S3. Paneth cells are not altered in GSD patients**

Determination of Paneth cells number per crypt in control and GSD patients by lysozyme-immunofluorescence staining. We evaluated the Paneth cells in formalin-fixed paraffin-embedded tissues samples from duodenal mucosa of GSD (n=6, black bars) and control subjects (N=3, white bars).


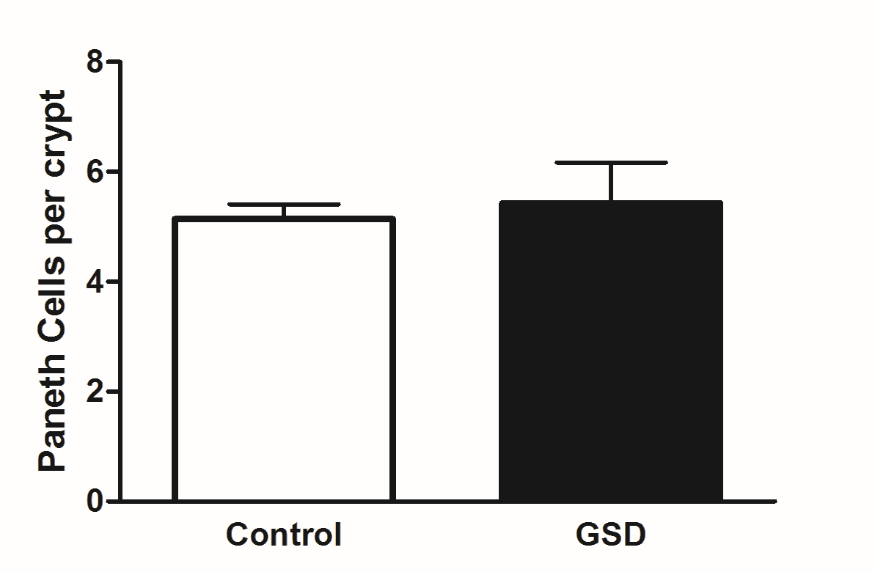


**Figure S4. qRT-PCR of differentially expressed genes related to apoptosis, epithelial tube morphogenesis, DNA replication, and phospholipid metabolic process.** We evaluated gene expression by qPCR in healthy volunteers and patients with GSD. CASP8, CASP9, BIRC3, and BCL2 were associated with apoptosis pathway; HIF1A, LPIN3, and PDX1 were involved in phospholipid metabolic process; OLFM4, LRG5, and WNT11 were associated with epithelial tube morphogenesis; CHEK1 were involved in DNA replication; and GUCA2A is a endogenous activator of intestinal guanylate cyclase. White dots represent healthy volunteers and black dots represent GSD patients. *18S* gene was used as normalizer. *p<0.05 and **p<0.01.

**Table S3. Primer list for real-time PCR**

| Gene | Primers | Sequences |
| --- | --- | --- |
| 18S | Forward Primer | AGCCACCCGAGATTGAGCAATA |
|  | Reverse Primer | CGAATGGGGTTCAACGGGTTAC |
| NPC1L1 | Forward Primer | ATTCCAGCTGGAGCCGAGTGA |
|  | Reverse Primer | AAGGAACCACTTGCAGGATGACCA |
| ABCG5 | Forward Primer | GTCCGAAGCAATGTGCTAAAGGGT |
|  | Reverse Primer | GATTCACAGCGTTCAGCATGCCT |
| ABCG8 | Forward Primer | TAAACTTGAGCAGCCTGTGGACAGTG |
|  | Reverse Primer | GGTGAGGTTCCCGAGAGGCATTTTAT |
| CASP1 | Forward Primer | CCGCAAGGTTCGATTTTCATTTGAGC |
|  | Reverse Primer | AATGTCCTGGGAAGAGGTAGAAACATC |
| OCLN | Forward Primer | GCAGGAAGGTCAAAGAGAACAGAGCA |
|  | Reverse Primer | TAGGTGGATATTCCCTGATCCAGTCCTC |
| TJP1 | Forward Primer | CCGAGGGATAGAAGTGCAAGTAGAGAGA |
|  | Reverse Primer | TCCGGGATTTCACCAGTGTGACTTTAG |
| REG3G | Forward Primer | ATGGATGGGAGTGGAGTAGCACTGAT |
|  | Reverse Primer | GCAGACATAGGGTAACTTTGCATCACAG |
| MT1M | Forward Primer | AACTGCTCCTGCACCACTGGTGT |
|  | Reverse Primer | TCCAACGTCCCTTTGCAGACAC |
| MT1E | Forward Primer | AACTGCTCTTGCGCCACTGGT |
|  | Reverse Primer | TTCTCCGATGCCCCTTTGCAG |
| DEFA6 | Forward Primer | GCAGAGGATGCAAGCTCAAGTCTTAG |
|  | Reverse Primer | CCCATGACAGTGCAGGTCCCATA |
| LCN2 | Forward Primer | GTGAGCACCAACTACAACCAGCAT |
|  | Reverse Primer | TTCCGAAGTCAGCTCCTTGGTTCT |
| CCL20 | Forward Primer | CAAGAGTTTGCTCCTGGCTGCTTT |
|  | Reverse Primer | TGCCGTGTGAAGCCCACAATA |
| CCR6 | Forward Primer | CTTGTGACGGCTGCAAATTTGGGTA |
|  | Reverse Primer | AAAAGCGTAGAGCACAGGGTTCAG |
| CHEK1 | Forward Primer | TGTGTCAGAGTCTCCCAGTGGATTTTC |
|  | Reverse Primer | GGGCTGGTATCCCATAAGGAAAGAC |
| GUCA2A | Forward Primer | CAGGGTTGGGAAACTCAGGAACTTTG |
|  | Reverse Primer | CTGGAAAGTTCGGGTTGCTACAGA |
| LPIN3 | Forward Primer | CAGAAGTCCTGAGCAGTGATGACGAT |
|  | Reverse Primer | AGGGACTTCTTGTAGGTAGGAGTGGA |
| HIF1a | Forward Primer | TGACCAGCAACTTGAGGAAGTACCAT |
|  | Reverse Primer | TTTCAGCGGTGGGTAATGGAGACA |
| BCL2 | Forward Primer | GAGGATTGTGGCCTTCTTTGAGTTCG |
|  | Reverse Primer | TGCCGGTTCAGGTACTCAGTCAT |
| CASP8 | Forward Primer | CGCAAAGGAAGCAAGAACCCATCA |
|  | Reverse Primer | GCAGCTCCTTCAGGAAGGACAGATTG |
| CASP9 | Forward Primer | AAGAGTGGCTCCTGGTACGTTGA |
|  | Reverse Primer | AACAGCATTAGCGACCCTAAGCAG |
| BIRC3 | Forward Primer | CAGACAGCCCAGGAGATGAAAATGC |
|  | Reverse Primer | CCATTTCCACGGCAGCATTAATCAC |
| OLFM4 | Forward Primer | CCACACTTCAGGTGCTAAACACTTG |
|  | Reverse Primer | TGGTGTTCATAGTACGGGTGGCAT |
| LGR5 | Forward Primer | CAATTTGGACAAGGGAGACCTGGAGA |
|  | Reverse Primer | AAGAAAGCCACAGGGCAGTTTAGGA |
| WNT11 | Forward Primer | CTGTGAAGGACTCGGAACTCGTCTAT |
|  | Reverse Primer | GTCGCTTCCGTTGGATGTCTTGTT |
| PDX1 | Forward Primer | GCTGCCTTTCCCATGGATGAAGTCTA |
|  | Reverse Primer | CGCGTCCGCTTGTTCTCCTC |

**Table S4. Gene expression of Zinc transporter in duodenum of Control and GSD subjects.**

| **Symbol** | **Control** | **GSD** | **padjusted** |
| --- | --- | --- | --- |
| ZIP1 | 621.3 ± 74.8 | 599.5 ± 63.2 | 0.862 |
| ZIP2 | 0 | 0 | nd |
| ZIP3 | 110.8 ± 22.5 | 108.2 ± 14.7 | 0.932 |
| ZIP4 | 2772.7 ± 1138.21 | 2386.2 ± 646 | 0.714 |
| ZIP5 | 3826.5 ± 224.4 | 3192.5 ± 267.6 | 0.059 |
| ZIP6 | 165.8 ± 17.4 | 175.5 ± 21.2 | 0.814 |
| ZIP7 | 1207.7 ± 56.9 | 1333.9 ± 113.87 | 0.382 |
| ZIP8 | 117.7 ±15.9 | 150 ± 24.9 | 0.232 |
| ZIP 9 | 1178.3 ± 117.1 | 1251.6 ± 118.3 | 0.702 |
| ZIP10 | 80.3 ± 14.9 | 112.9 ± 5.4 | 0.059 |
| ZIP11 | 455.7 ± 49.5 | 665 ± 108.6 | 0.014 |
| ZIP12 | 0 | 0 | nd |
| ZIP13 | 189 ± 10.9 | 168.4 ± 22.5 | 0.503 |
| ZIP14 | 6507.7 ± 431.7 | 6619.3 ± 1235.7 | 0.951 |
| ZNT1 | 1530.7 ± 143.3 | 1878.8 ± 382.7 | 0.263 |
| ZNT2 | 0 | 0 | nd |
| ZNT3 | 0 | 0 | nd |
| ZNT4 | 531.9 ± 61.1 | 658.3 ± 160.8 | 0.351 |
| ZNT5 | 622.1 ± 16.6 | 687.5 ± 55.23 | 0.371 |
| ZNT6 | 341.2 ± 14.6 | 405 ± 30.9 | 0.126 |
| ZNT7 | 390.8 ± 16.2 | 438.8 ± 30.4 | 0.318 |
| ZNT8 | 20.7 ± 6.7 | 25.8 ± 7.8 | 0.648 |
| ZNT9 | 887 ± 113.7 | 964 ± 120 | 0.648 |
| ZNT10 | 966.8 ± 259.2 | 872.2 ± 262.7 | 0.783 |

nd = no determined
